# Distributional Benefit Analysis of a National Air Quality Rule

**DOI:** 10.3390/ijerph8061872

**Published:** 2011-06-01

**Authors:** Ellen S. Post, Anna Belova, Jin Huang

**Affiliations:** Environment and Resources Division, Abt Associates Inc., 4550 Montgomery Avenue, Suite 800 North, Bethesda, MD 20814, USA; E-Mails: ellen_post@abtassoc.com (E.S.P.); jin_huang@abtassoc.com (J.H.)

**Keywords:** distributional analysis, environmental justice, air quality regulation, health benefits

## Abstract

Under Executive Order 12898, the U.S. Environmental Protection Agency (EPA) must perform environmental justice (EJ) reviews of its rules and regulations. EJ analyses address the hypothesis that environmental disamenities are experienced disproportionately by poor and/or minority subgroups. Such analyses typically use communities as the unit of analysis. While community-based approaches make sense when considering where polluting sources locate, they are less appropriate for national air quality rules affecting many sources and pollutants that can travel thousands of miles. We compare exposures and health risks of EJ-identified individuals rather than communities to analyze EPA’s Heavy Duty Diesel (HDD) rule as an example national air quality rule. Air pollutant exposures are estimated within grid cells by air quality models; all individuals in the same grid cell are assigned the same exposure. Using an inequality index, we find that inequality within racial/ethnic subgroups far outweighs inequality between them. We find, moreover, that the HDD rule leaves between-subgroup inequality essentially unchanged. Changes in health risks depend also on subgroups’ baseline incidence rates, which differ across subgroups. Thus, health risk reductions may not follow the same pattern as reductions in exposure. These results are likely representative of other national air quality rules as well.

## 1. Introduction

“Environmental justice” (EJ) has become a pressing social, scientific, and political issue in the U.S. over the last decade. The 1994 Executive Order 12898 (*Federal Action to Address Environmental Justice in Minority Populations and Low-Income Populations*), requires agencies to perform EJ reviews of programs, policies, and activities in order to determine their effects on minority and low-income populations. The U.S. Environmental Protection Agency (EPA) defines “environmental justice” as “the fair treatment and meaningful involvement of all people regardless of race, color, national origin, or income with respect to the development, implementation, and enforcement of environmental laws, regulations, and policies.” EPA further defines “fair treatment” to mean that “no group of people should bear a disproportionate share of the negative environmental consequences resulting from industrial, governmental and commercial operations or policies.”

This definition provides very general guidance on the concept of EJ, but does not supply specifics and directions for applying this concept to EPA’s programs and activities. In this paper, we focus on the benefits of national air quality rules and propose a comprehensive set of methods that can be used to examine several EJ questions relevant to the context:

- Are different socio-demographic population subgroups being exposed to different pollution levels before a rule is implemented (baseline scenario)?
- When a given rule is implemented, do different subgroups benefit differentially? That is, do some subgroups enjoy greater reductions in pollution levels than others?
- Do some subgroups enjoy greater reductions in health risks as a result of a given rule or regulation?
- As a result of a given rule, do the pollutant exposures (and associated health risks) experienced by different subgroups become less unequal?

We use EPA’s Heavy Duty Diesel rule [1] (henceforth, the HDD rule) as a case study. There are several socio-demographic population subgroups that may be of interest from the EJ standpoint. EJ subgroups can be defined by age, sex, race, ethnicity, education, and/or income and should not overlap. We focus on race and ethnicity in our EJ analysis of the HDD rule, with the recognition that the method we describe could similarly be used with other categorizations.

Many studies have explored the validity of the EJ hypothesis that environmental disamenities are experienced disproportionately by poor and/or minority subgroups. The two most common types of EJ research are: (1) proximity-to-hazards studies and (2) exposure and health risk analysis. The first category evaluates how the distribution and proximity of hazards (e.g., Superfund sites, toxic emissions, and existing waste facilities) relate to community socio-demographics [2–6]. Residential proximity to a waste site or other hazards is often used as a surrogate measure for exposure to contaminants found at those sites. The second category of EJ research, exposure and risk analysis, examines the distributions of exposures and health risks among different EJ subgroups [7–12].

Regardless of whether they are proximity-to-hazards studies or exposure and health risk studies, most EJ analyses have taken as their unit of analysis some geographical measure of community, such as county [13,14], neighborhood [15], census tract [3,8,9,11,14], or zip code [16]. Recent studies have examined associations between a defined EJ measure (e.g., percentage of minorities in the community) and ambient pollution levels as well as health risks [8,9,11].

While community-based approaches make sense for considering where particular polluting (point) sources locate, national air quality rules target thousands of emissions sources and pollutants that can travel many miles. The ambient pollutant concentrations to which people are exposed depend on many possibly distant sources and complex air chemistry. This is true of fine particulate matter (particles with a mean aerodynamic diameter less than or equal to 2.5 μm, denoted PM_2.5_) analyzed in the HDD rule. PM_2.5_ can form so-called “secondary” reactions in the atmosphere, many miles from the original sources of the precursor emissions. Thus, the issue of location decisions by particular emissions sources is less relevant. In this context, the questions of primary interest are whether the members of one EJ subgroup are exposed to higher ambient pollutant concentrations as compared to the members of other subgroups, and whether a national air quality rule will benefit some subgroups disproportionately. Communities—whether they are defined as census tracts, counties, zip codes, or any other unit of geographic area—are artificial analytical constructs that are not necessary to answer these questions. In fact, we are interested in *all* African Americans, regardless of where they live, compared with *all* Whites, *all* Hispanics, *etc.*

An individual-level conceptualization permits application of methods that are well tailored for exploration of the EJ questions relevant to benefits assessment of national air quality rules. The central object of analysis for these methods is an empirical distribution of the quantity of interest (e.g., exposure) over *all* individuals belonging to a given subgroup. Individual-level air pollution exposures and health risks have previously been used to construct inequality indices for examining efficiency-equity tradeoffs in air quality control policies [10,17,18], and to compare distributions of exposures to toxic air emissions among EJ subgroups [13].

We extend the methods in these papers to demonstrate the insights that may be gained about the EJ questions relevant to benefits assessment of national air quality rules by carrying out a *distributional analysis* of exposures and health risks. This analysis consists in comparing EJ subgroup-specific distributions over individuals. Information contained in empirical distributions permits a broader assessment of differences among subgroups in exposures and health risks. Because exposures and health risks are highly variable, an analysis that explores associations between central tendencies (as many community-based analyses tend to do) would miss out on many interesting and important insights. For instance, we can analyze the differences in exposures between Whites and African Americans at the 95th percentile of the distribution of exposures. This comparison would be impossible through a community-based analysis that focuses on correlations between exposure or health risk levels and aggregate community characteristics (e.g., median household income or the proportion of African Americans).

It is not feasible to measure individual-specific air pollution exposures for benefits assessments of national air quality rules: such assessments are generally conducted for a *future* year and involve *hypothetical* policy scenarios. Therefore, they use *modeled* exposures rather than *measured* exposures to the ambient air pollutant. Air quality models generate estimates of pollutant concentrations on a grid that spans the entire country. All individuals *projected* to reside within the same grid cell are assigned the same air pollutant concentration [1,19]. We follow this procedure. Because people are mobile, a modeled grid cell-level air pollutant concentration may provide a reasonable approximation to average individual-specific exposures.

However, there will always be some degree of uncertainty about actual exposures whenever modeled data and projections are used. Furthermore, regardless of the analytical unit chosen (an individual or a community), any analysis that uses modeled ambient air pollutant concentrations is limited by the spatial resolution of the model.

Although EPA refers to “fair treatment,” observed differences in exposures to air pollutants on a national or regional level do not necessarily imply *unfair* treatment in the normal sense of that term—*i.e.*, unfair intent. While air pollutants are generated to some extent by stationary sources (e.g., power plants), where someone had to decide where to locate the source of pollution, these pollutants can travel great distances. The juxtaposition of population subgroups relative to areas of poor air quality may also reflect the choices people make of where to live. In general, it is more difficult to discern the *why* of any observed differences among subgroups for regional air pollutants than for local pollutants. The methods that we propose for distributional benefits analyses of national air quality rules are not intended to answer the question of why there are differences in the levels of air pollution to which different subgroups are exposed, but only whether there are differences.

## 2. Distributional Benefits Analysis of EPA’s Heavy Duty Diesel Rule in 2030

EPA’s HDD rule, published in 2001, is a part of EPA’s comprehensive national control program to regulate the heavy-duty vehicle and its fuel as a single system [1]. The HDD rule included new standards for particulate matter (PM), the oxides of nitrogen (NOx), and non-methane hydrocarbon (NMHC) emissions by heavy-duty highway engines and vehicles. Because the exhaust emission control devices required by the HDD rule could be damaged by sulfur, EPA also regulated refiners and the fuel distribution chain to make diesel fuel with low sulfur content available for highway vehicles.

This nationwide program is expected to result in emission levels of PM and NOx that are 90 percent and 95 percent, respectively, below the current National Ambient Air Quality Standards for these air pollutants [20]. EPA analyzed the expected benefits of this rule in the years 2020 and 2030. We use the modeled air quality data for 2030.

We define our EJ subgroups in terms of race and ethnicity. Following the nomenclature of the U.S. Census Bureau, we consider four racial subgroups—Asian American, African American, Native American, and White—as well as two ethnic subgroups (Hispanic and non-Hispanic). For individuals in each racial subgroup as well as for individuals in (mutually exclusive) subgroups defined by combinations of racial, ethnic, and age characteristics, we examine:

- The ambient PM_2.5_ concentrations to which they are expected to be exposed in the 2030 baseline (*i.e.*, in the absence of the rule);
- The reductions in ambient PM_2.5_ concentrations they are expected to experience in 2030 as a result of the rule; and
- The corresponding reductions in all-cause mortality they are expected to experience as a result of the rule.

### 2.1. Estimating Baseline Pollutant Exposures and Reductions in Exposure as a Result of the HDD Rule

Our distributional analysis of the HDD rule consists in carrying out what is essentially a standard EPA benefit analysis separately for each subgroup and then comparing the subgroup-specific results. For national air quality regulations, EPA relies on the Environmental Benefits Mapping and Analysis Program (BenMAP) to estimate the health impacts and economic benefits associated with changes in ambient air pollution [21,22]. The changes in air pollution are typically calculated with the help of air quality models that use air pollution emissions data and meteorological data in a complicated series of calculations representing the formation and movement of air pollution in the atmosphere. Air quality modeling is necessary because it can provide estimates of air pollution levels in areas of the country where actual air pollution monitoring data are not available (e.g., rural areas) and because it can generate projections of air pollution levels for hypothetical policy scenarios.

**Figure 1.**
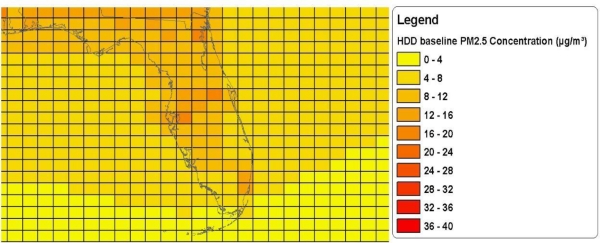
A Portion of the Baseline Air Quality Grid (Over Florida) Used in the HDD rule Benefit Analysis. *Source:* U.S. EPA Final Regulatory Impact Analysis: HDD rule.

Air quality models calculate air pollution levels separately for each cell in a grid that spans the country. Figure 1 shows a portion of the grid with the baseline air quality estimates used in the HDD rule. The grid cells for that analysis were roughly 36 kilometers by 36 kilometers.

More recent national analyses, such as the EPA’s Locomotive and Marine Rule [19], are using grid cells that are roughly 12 kilometers by 12 kilometers. In general, the resolution of air quality models is increasing over time due to improvements in data processing ability. It is currently possible to have even more refined analyses with grid cells that are 1 kilometer by 1 kilometer (or smaller) for local analyses, such as analyses of individual metropolitan areas.

**Figure 2.**
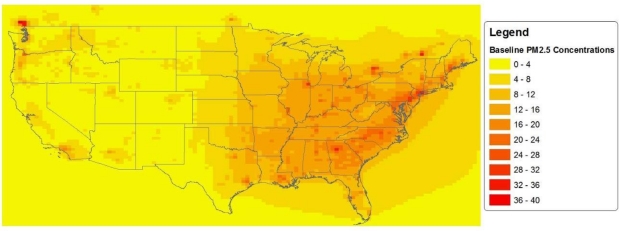
Projected 2030 Baseline Annual Average Ambient PM_2.5_ Concentrations (μg/m^3^).

**Figure 3.**
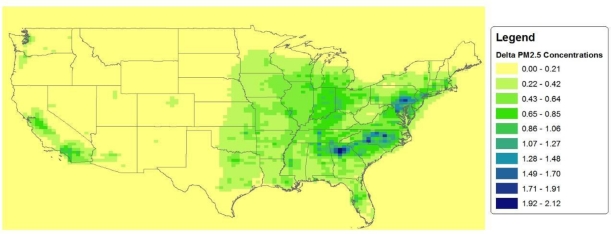
Projected 2030 Reductions in Annual Average Ambient PM_2.5_ Concentration (μg/m^3^).

Figure 2 shows a map of projected annual average ambient PM_2.5_ concentrations in the 2030 baseline (*i.e.*, without the regulation). The air quality data files for the HDD rule also contain projected annual average ambient PM_2.5_ concentrations in the 2030 control scenario: the scenario of future conditions with the regulation in place (*i.e.*, after air pollution controls have been implemented). For each grid cell we calculate the reduction in ambient PM_2.5_ concentration (that is projected to occur as a result of the HDD rule) as the difference between the baseline and the control scenario modeled PM_2.5_ concentrations. Figure 3 shows the projected reductions in ambient PM_2.5_ concentrations across the U.S. Note that, for regions that have high baseline PM_2.5_ concentrations, the reductions expected to result from the HDD rule are also relatively large. This indicates that the HDD rule tends to target the most polluted areas.

The version of BenMAP used for this distributional analysis as well as for the original benefits assessment of the HDD rule relies on 2000 Census of Population and Housing block-level data. BenMAP uses the embedded population growth projections for EJ subgroups [23] to extrapolate the 2000 EJ subgroup-specific population counts in each block to 2030, the analysis year. The procedures used to create the projections are detailed in the BenMAP user manual [21].

**Figure 4.**
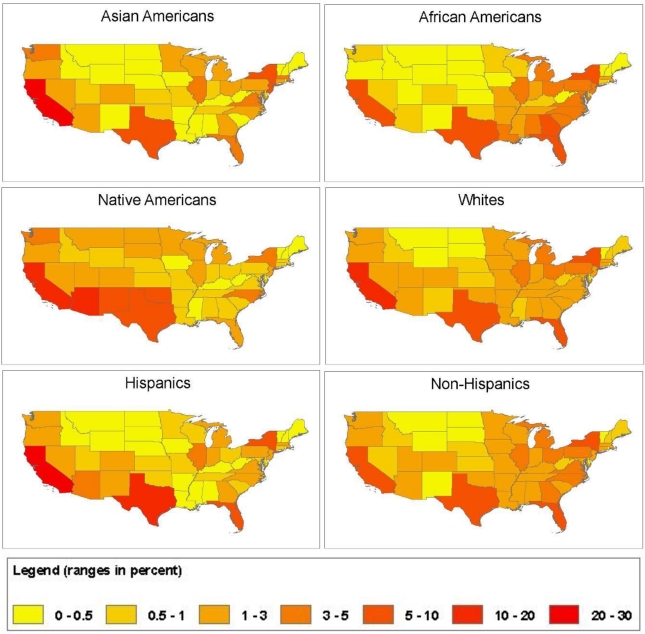
Projected 2030 Spatial Population Distributions for EJ Subgroups by State.

Figure 4 shows maps of EJ subgroup-specific projected population distributions by state. A comparison of the projected population distribution maps (Figure 4) with the map of projected baseline ambient PM_2.5_ concentrations (Figure 2) gives a “broad brush” picture of which EJ subgroups are projected to live in areas of high *versus* low PM_2.5_ concentrations. For example, the high PM_2.5_ concentrations are mostly in the Eastern half of the U.S. and, to a lesser extent, in southern California—areas in which most African Americans and Asian Americans are projected to live. In contrast, Native Americans are projected to be concentrated largely in the Southwest, where projected PM_2.5_ concentrations are low, and to some extent in California.

Air quality model grid cells typically cross territorial units that the U.S. Census Bureau defines for purposes of taking a population census. To calculate the EJ subgroup-specific projected 2030 population in each grid cell, BenMAP aggregates projected census block population data. Although census blocks vary in size, 99 percent are smaller than 4 kilometers by 4 kilometers, which is much smaller than the grid cells in all but the highest resolution grids. Thus, a grid cell will have several to many census blocks that fall completely within it. For those census blocks that straddle two or more grid cells, BenMAP assigns the block population to the grid cell in which the census block center is located.

The modeled ambient PM_2.5_ concentration in each grid cell is then assigned to all individuals in that grid cell. Reductions in ambient PM_2.5_ concentrations due to the HDD rule are similarly assigned. All individuals are identified by the EJ subgroup to which they belong. We use the projected data on EJ subgroup-specific 2030 population counts and modeled PM_2.5_ concentrations in each grid cell to construct empirical distributions of pollutant exposures over individuals in each EJ subgroup. Empirical distributions of reductions in PM_2.5_ concentrations due to the HDD rule over individuals in EJ subgroups are constructed analogously.

**Figure 5.**
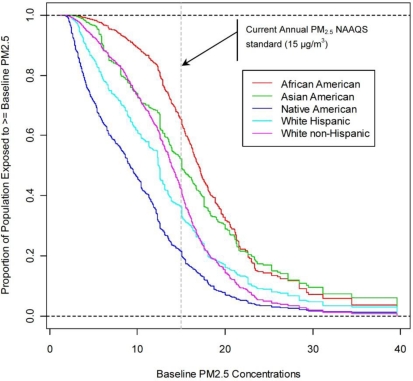
Racial and Ethnic Subgroup-Specific Distributions of 2030 Projected Baseline PM_2.5_ Concentrations.

**Figure 6.**
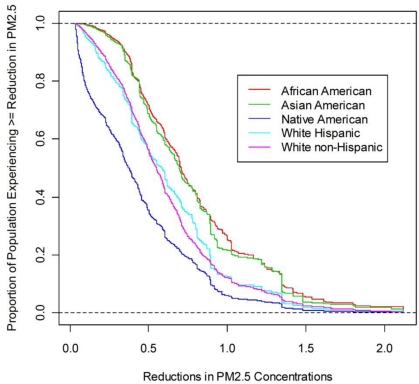
Racial and Ethnic Subgroup-Specific Distributions of 2030 Projected Reduction in PM_2.5_ Concentrations.

Figures 5 and 6 show EJ subgroup-specific cumulative empirical distributions of baseline PM_2.5_ concentrations and reductions in PM_2.5_ concentrations as a result of the HDD rule, respectively. In Figure 5, any point (x, y) along each cumulative distribution shows that 100*y percent of that EJ subgroup is exposed to more than *x* μg/m^3^ baseline PM_2.5_ concentration. Figure 6 is interpreted similarly. In both cases, the closer the cumulative distribution is to a vertical line, the less inequality there is in exposures (or reductions in exposure) among individuals in the subgroup. We can see immediately that for any baseline PM_2.5_ concentration, the percent of Native Americans exposed to at least this level is the smallest across all subgroups. The opposite is true for African Americans for baseline concentrations up to about 25 μg/m^3^. Greater percentages of Asian Americans are exposed to the highest baseline PM_2.5_ concentrations. About 65 percent of African Americans are projected to be exposed to ambient PM_2.5_ concentrations in excess of the current National Ambient Air Quality Standard (NAAQS) for annual average PM_2.5_ (15 μg/m^3^) [24]; only about 20 percent of Native Americans are projected to live in areas with exposures that exceed the standard. In the remaining subgroups, the corresponding shares are between 35 and 55 percent.

Figure 6 shows that the distributions of absolute reductions in exposure levels due to the HDD rule largely mimic the distributions of baseline exposures themselves. For instance, Native Americans have the lowest reductions and the lowest baseline exposure levels, while African Americans and Asian Americans have the highest reductions as well as the highest baseline exposure levels. The similarity in patterns shown in Figures 5 and 6 implies that all the subgroups might enjoy similar relative reductions in exposure levels. That is, the likely effect of the HDD rule would be to scale down the baseline exposure levels by approximately the same factor.

Characteristics of the distributions shown in Figures 5 and 6—the means, standard deviations, and selected percentiles—are given in Tables 1 and 2, respectively. Table 2 shows these characteristics for both the absolute reductions in PM_2.5_ levels and the relative reductions, that is, the percent reductions from baseline levels.

Tables 1 and 2 also show the results of applying an inequality index to the empirical distributions of baseline PM_2.5_ exposures as well as of reductions in these exposures. This index characterizes the degree of inequality by assigning a single score to the distribution [25]. We use the Atkinson index, which was proposed by Levy *et al.* as the most appropriate inequality index for health risk analysis [17]. The Atkinson index is derived from a social welfare function [26]. It depends on an inequality aversion parameter, *ɛ* > 0. When *ɛ* < 1, the Atkinson index is more sensitive to changes in the top of the distribution. When *ɛ* > 1, the index is more sensitive to changes in the bottom of the distribution. This index has a maximum of 1, which indicates extreme inequality, and a minimum of 0, which indicates absolute equality. We report the Atkinson index for *ɛ* = 0.5 and *ɛ* = 1.

The Atkinson index is decomposable, which allows us to determine the extent to which the total inequality is attributable to inequality within non-overlapping population subgroups *versus* between them. Informally, a decomposable inequality index (*I*) can be computed as a combination of inequality indices for each subgroup (*within-group* inequality, *I_w_)* and an inequality index that represents disparities across subgroups (*between-group* inequality, *I_b_*). The between-group inequality is computed over arithmetic means of the subgroup-specific distributions. A formal definition of decomposability is provided by Cowell [27]. Although the Atkinson index can be decomposed, this decomposition is not additive: (1 – *I*) = (1 – *I_w_*)·(1 – *I_b_*) [28].

Table 1 shows that Native Americans are exposed to lower baseline PM_2.5_ levels, whereas African Americans and Asian Americans are exposed to higher baseline PM_2.5_ levels (as is also shown in Figure 5). The general patterns seen in the subgroup-specific means are also seen in the distributions as a whole. While African Americans and Asian Americans have the highest mean baseline PM_2.5_ concentrations, for example, they also have the highest 75th and 95th percentile concentrations.

The inequality index results, however, put the differences across subgroup-specific means and percentile values in a broader context. Table 1 shows that within-subgroup inequality in baseline PM_2.5_ exposures is much greater than between-subgroup inequality. This is true for control scenario exposures as well. For *ɛ* = 0.5, the ratio of within-group inequality to between-group inequality is 15 in both the baseline and the control scenario; for *ɛ* = 1, this ratio is 13.7 in both scenarios. Thus, the differences between subgroup-specific means and percentiles that we see in Table 1 seem much less substantial. The lack of change in the inequality index from the baseline to the control scenario, for either choice of *ɛ*, indicates that inequality in exposures among subgroups was unaffected by the HDD rule. The picture that emerges is one of relatively low inequality among subgroups in the baseline (as compared to within-subgroup inequality) and consistently decreasing exposures across subgroups as a result of the rule, rather than decreasing *inequality of* exposures across subgroups.

For both choices of *ɛ*, we see that Native Americans have more inequality than any other subgroup under both the baseline and the control scenario, and African Americans have the lowest inequality. Combined with the distribution characteristics, this creates a picture of a relatively wider range of exposures among Native Americans, generally weighted towards the low end of the range of exposures, and a relatively narrow range of exposures among African Americans up towards the high end of the range of exposures. These patterns reflect the spatial distributions of these two subgroups. A high proportion of Native Americans lives in areas with very low levels of PM_2.5_ (*i.e.*, in the Southwest and in the Northern Great Plains), while a small proportion lives in urban areas with high concentrations of air pollution. In contrast, most African Americans live in urban areas.

Table 2 shows that Native Americans are predicted to experience substantially smaller absolute reductions in PM_2.5_ levels than the other subgroups, while African Americans and Asian Americans are predicted to experience larger absolute reductions. Again, however, only a relatively small share of the total inequality in absolute reductions in PM_2.5_ exposures among individuals in the total population is due to inequality across subgroups, for either choice of *ɛ*. The ratio of within-group inequality to between-group inequality in absolute reductions is 21.3 for *ɛ* = 0.5 and 17.5 for *ɛ* = 1; the corresponding ratios for relative reductions are 29.0 and 29.5, respectively.

The relative reductions, shown in Table 2, are very similar across subgroups, on average. The values of the Atkinson index for relative reductions also suggest that there is very little variability in relative reductions of exposure. Given that the Atkinson index is scale-invariant, essentially identical relative reductions in exposure would not affect inequality in exposure levels. Thus, the results in Table 2 corroborate the finding, shown in Table 1, that the HDD rule had little effect on the degree of inequality within and between subgroups.

Although there is uncertainty about our results, there is no uncertainty due to sampling error surrounding the estimates in Tables 1 and 2, because these distribution characteristics are not based on samples but on a complete census of the population. Thus, the usual tests to determine whether estimated means are statistically significantly different from each other do not apply.

### 2.2. Estimating Reductions in Health Effect Incidence Rates Corresponding to Reductions in PM_2.5_

Exposure to PM_2.5_ has been associated with several adverse health effects, including premature mortality, non-fatal acute myocardial infarction, emergency room visits for asthma, and cause-specific hospital admissions [29–34]. Here we focus on premature mortality, but the approach described below would be similar for all other health effects.

We use two estimated concentration-response (C-R) relationships describing the association between PM_2.5_ concentrations and premature mortality: one for infants [33] and another for adults age 30 and older [34]. The epidemiological literature does not currently provide estimated C-R relationships for PM_2.5_ concentrations and premature mortality for ages 1–29. Because the available C-R relationships are not EJ subgroup-specific, our analysis implicitly assumes that the C-R relationship is the same across EJ subgroups. Both C-R relationships have the exponential form:

(1) $$y=\alpha *e^{\beta x}$$

where *x* is the ambient PM_2.5_ level, *y* is the incidence of mortality corresponding to *x*, *β* is the coefficient of ambient PM_2.5_ concentration (describing the extent of change in *y* with a unit change in *x*), and parameter *α* is the incidence when there is no ambient PM_2.5_. Each epidemiological study provides *b*—an estimate of *β*.

Let *x*_0_ denote the baseline (upper) level of ambient PM_2.5_ and *x*_1_ denote the control scenario (lower) level of ambient PM_2.5_. In addition, let *y*_0_ denote the baseline incidence (corresponding to the baseline ambient PM_2.5_ level, *x*_0_) and *y*_1_ denote the incidence after the rule is implemented. Equation (1) can be used to derive the following relationship between the absolute reduction in ambient PM_2.5_ level, Δ*x* = (*x*_0_ – *x*_1_), and the corresponding reduction in mortality incidence, Δ*y*:

(2) $$\Delta y=(y_{0}-y_{1})=y_{0}\cdot (1-e^{-b\cdot \Delta x})$$

We use Equation (2) to estimate the reductions in mortality incidence in each grid cell. Baseline mortality incidence *y*_0_ is calculated as the product of the mortality incidence rate and the population in each grid cell. We derive baseline mortality incidence rates from county-level mortality data for 1996–1998 provided by the U.S. Centers for Disease Control (CDC). Age range-specific death count data are available for three race subgroups: White, African American, and “other.” Having calculated age- and county-specific mortality rates for these three race subgroups [21], we assign the “other” mortality rate to the Native American and Asian American subgroups. Baseline mortality rates are projected to 2030 [21]. Given that CDC does not provide mortality data by ethnicity, in this section we consider EJ subgroups defined by race only.

Table 3 presents the reduction in mortality incidence rate alongside the reduction in PM_2.5_ concentration that each racial subgroup is predicted to experience as a result of the HDD rule. This juxtaposition makes it easier to see the correspondence or lack thereof between the two. For each racial subgroup, we consider three age categories: infants (age 0), adults (age 30–64), and elderly (age 65+). We show the mean absolute reduction in PM_2.5_ exposure that each age/ racial subgroup is predicted to experience as well as the mean absolute reduction in mortality rate. We also show the reduction in PM_2.5_ exposure relative to the mean reduction for the total population in the age category and the corresponding relative reduction in mortality rate. The relative reduction allows us to see at a glance how one subgroup is expected to fare relative to others, in terms of both the reduction in PM_2.5_ concentration and mortality rate.

Asian Americans are predicted to experience about 20 percent greater PM_2.5_ exposure reductions, on average, than the total population (*i.e*., a relative reduction of 1.2), while African Americans are predicted to experience from 20 percent to 30 percent greater PM_2.5_ exposure reductions, on average, depending on the age subgroup considered. Native Americans, on the other hand, are predicted to experience smaller reductions in PM_2.5_ exposure than the total population, on average—about 70 percent of the reduction for the total population. Finally, Whites are predicted to experience reductions in PM_2.5_ exposure that are basically the same as those of the total population (relative reduction of 0.9 for infants and ages 30–64, and 1.0 for the elderly).

As shown in Table 3, the relative reductions in PM_2.5_ exposure predicted to be enjoyed by the different racial subgroups do not necessarily translate into the same relative reductions in mortality. This is because the reductions in mortality depend, in addition, on the baseline mortality incidence rates, and these differ substantially across the racial subgroups. For example, both African American and Asian American infants are predicted to experience about 20 percent greater reductions in PM_2.5_ exposure than the total population of infants, on average. However, because the mortality rate among African American infants is so much higher than that among Asian American infants (9,543 *vs.* 2,907 deaths per million), African American infants are predicted to experience a much greater relative reduction in mortality rate than Asian American infants (over 230 percent *versus* only 70 percent). This reflects the greater underlying vulnerability of African American infants, relative to the general population (or, for that matter, to any other subgroup).

Table 4 characterizes distributions of mortality risk reductions due to the HDD rule over individuals in subgroups defined by race and age. For *ɛ* = 0.5, the ratio of within-group inequality to between-group inequality in mortality risk reductions is 1.6 for infants and 3.0 for adults. In the case of the distribution of absolute reductions in PM_2.5_ exposure, this ratio is much higher: with identical choice of *ɛ* it is 21.3 (reported in Table 2). Thus, in the case of mortality rate reductions, differences among racial subgroups tend to contribute more to overall inequality. This, of course, is largely driven by the across-subgroup differences in baseline mortality incidence rates that reflect the underlying vulnerabilities of these populations and may have little to do with the effects of the HDD rule.

## 3. Discussion

EJ analyses were originally developed to address a common hypothesis that environmental disamenities locate disproportionately in poor or predominantly minority communities in part because of the socio-demographic makeup of those communities. Executive Order 12898 later mandated that federal agencies carry out EJ reviews of their programs, rules, and regulations. National air quality rules present a different set of issues from the location issues originally posed. As noted above, observed inequalities in air pollutant exposures at a regional or national level do not necessarily imply injustice in the normal sense of that word. While air pollutants are generated to some extent by stationary sources (e.g., power plants), where someone had to decide where to locate the source of pollution, these pollutants can travel great distances and can be formed by reactions in the atmosphere, many miles from the original sources of the precursor emissions. This is an important consideration, particularly in interpreting the results of a distributional benefits analysis of national air quality regulations. If we see differences in pollutant concentrations to which the members of one subgroup are exposed *versus* those in other subgroups, it does not necessarily follow that these differences are the result of unfair intent.

The juxtaposition of subpopulations relative to areas of poor air quality may also reflect the choices people make of where to live. The location of poorer individuals in areas of higher pollution may, to some extent, reflect tradeoffs made by these individuals—*i.e*., some may choose to live in higher pollution areas if the housing there is more affordable [35]. Residential location decisions may also reflect the historical patterns of settlement of different ethnic subgroups coming to the U.S. over time. For example, Asian Americans historically settled disproportionately in large urban areas [36,37], where traditional “ports of entry” were located. More than 60 percent of Asian Americans live in eight large metropolitan areas and more than 40 percent of Asian Americans live in New York, Los Angeles, and San Francisco metropolitan areas [37]. Coincidentally, these areas also have relatively poor air quality (Figure 1). Thus, the exposure of Asian Americans to relatively poorer air quality may reflect the effects of national immigration policies that regulated settlement patterns [38].

The distributional benefits analysis method we propose for national air quality rules is intended to answer the question of whether there are differences, but not why there are differences in the levels to which various subgroups are exposed. Similarly, we are not asking why different subgroups may benefit differentially from a rule or regulation, but simply whether or not they do benefit differentially—in terms of the reductions in air pollution they experience as a result of the rule and in terms of the health risk reductions they enjoy as a result of the reductions in air pollution.

However, because there are differences in pollutant exposures (or reductions in exposures as a result of a rule) among individuals *within* subgroups, the question of whether there are differences *between* subgroups is best answered by a comparison of subgroup-specific distributions of exposures (or reductions in exposures) over individuals. We believe that such an approach, discussed and illustrated above, generates more interesting insights about the context-relevant EJ questions than those obtainable with a community-based approach. Using subgroup-specific distributions, we can get a fuller picture of inequality (or lack thereof) both between subgroups and within them. We saw, for example, that the inequality in baseline PM_2.5_ concentrations predicted to be experienced by different subgroups, as illustrated by comparisons of their means, is very small compared with the inequality of exposures within subgroups, as shown by decomposition of the Atkinson index (Table 1). We saw also that, while both African Americans and Asian Americans are predicted to experience higher baseline PM_2.5_ concentrations than the other subgroups, greater proportions of Asian Americans are predicted to experience the highest baseline PM_2.5_ concentrations of over about 25 μg/m^3^ (Figure 5).

We saw that, for a national air quality rule, those subgroups that are exposed to higher baseline pollutant concentrations, on average, tend to enjoy greater absolute reductions in pollutant concentrations as a result of the rule. This is not surprising, since many rules tend to target the areas of worst pollution levels. Furthermore, all EJ subgroups experience similar relative reductions in baseline exposures. As a result, neither the between-group inequality nor within-group inequality would be affected by the HDD rule.

We also saw that the reduction in air pollutant concentrations did not necessarily translate into an equivalent reduction in health effect incidence rate in the different subgroups—e.g., the subgroup that experiences the largest reduction in pollutant concentration as a result of a rule does not necessarily also experience the largest reductions in incidence rates of adverse health effects associated with the pollutant. This is because another factor—the baseline incidence rate of the adverse health effect— affects each subgroup’s population health response to a reduction in pollutant concentration, and these baseline incidence rates vary substantially across racial and ethnic subgroups.

We note also that the type of distributional analysis we describe addresses only one of several possible distributional effects of an air quality rule or regulation, *i.e.*, the distribution of benefits across defined subgroups. Fullerton describes several types of distributional effects, *i.e.*, price changes, scarcity rents, benefits effects, capitalization effects, and transition costs [39]. While, in theory, one should consider all distributional effects together to get the “full picture,” in practice that would be very difficult to do. Although there are several kinds of distributional effects that could occur, we are not aware of any empirical paper that actually includes all of these effects or even most of them.

As we note above, in the assignment of pollutant concentrations, or reductions in pollutant concentrations as a result of the HDD rule, our analysis could only approximate an individual-level analysis, because modeling truly individual-specific pollutant exposures is not feasible. Available air quality models convert projected emissions from various sources to ambient pollutant concentrations in cells of a grid that spans the country. As is done in standard analyses of national air quality rules, we assign the same baseline (and control) scenario pollutant concentration to all individuals within a grid cell. Whether this method of estimating exposures for individuals is adequate depends on the extent of intra-grid cell variability in pollutant concentrations. This is likely to be less of a problem for regional pollutants, such as PM_2.5_ and ozone, than for more local pollutants, such as carbon monoxide, whose concentrations tend to vary more within any given grid cell. Analysis of mobile source rules, such as the HDD rule, may pose a particular challenge, because these rules target pollutant sources along transportation corridors within grid cells. It is unclear to what extent this pollution dissipates, and if so, how quickly.

Because intra-grid cell differences between subgroups are also obscured by this method of exposure assignment, there are special implications of using it in the context of an EJ analysis (be it individual-based or community-based). If intra-grid cell heterogeneity follows patterns that are dependent on an EJ characteristic, any approach that depends on grid cell-level pollutant estimates, may understate differences across EJ subgroups. This may partly explain why our distributional analysis of the baseline exposure levels and reductions in them (due to the HDD rule) finds low between-group inequality.

The accuracy of inequality assessment could be improved through increasing spatial resolution of the air quality models. However, because people are mobile, extremely small grid cell sizes will introduce other biases. The problem of the optimal grid cell size is shared by standard and EJ-oriented air quality benefits analyses. It would be instructive to progressively reduce the grid cell size in an EJ analysis and observe how it affects the results.

To assess whether pollution affects some subgroups disproportionately, some studies [8,40] have applied regression techniques and statistical tests to what appear to be complete censuses rather than random samples (e.g., all the census tracts in a given state), and have reported “statistically significant” results. “Statistical significance,” however, is a meaningful concept only when an analysis is based on a random sample (rather than the entire population of interest). “Statistical significance” suggests that what we observe in the sample indicates something real about the population, rather than being due to random chance (*i.e*., to the particular sample we randomly drew from the population). If we are observing the entire population (e.g., all the census tracts in a state), then we should not use statistical tests, as “statistical significance” is meaningless. This does not imply that there is no uncertainty in an EJ analysis that uses the entire population, only that there is no uncertainty due to sampling error associated with sampling from the population. All of the uncertainty in air quality benefits analyses— including the uncertainty surrounding air quality model estimates of exposure and population projections, as well as the uncertainty surrounding estimated concentration-response relationships—applies to the corresponding EJ analyses as well. Most of this uncertainty is difficult to quantify.

Even if there were no uncertainty in our results, there is a legitimate question as to what magnitude of differences between subgroups constitutes “environmental injustice.” Since it is highly improbable that all subgroups would have exactly the same baseline pollutant concentrations or reductions in pollutant concentrations, there will necessarily be differences between subgroups. Rather than “statistical significance,” the relevant question is whether observed differences between populations (e.g., between minorities and non-minorities) are worthy of concern. At what point should any observed differences be considered disproportionate? This is more likely a policy decision, rather than a question that economics can necessarily answer. There is no objective degree of difference beyond which we definitively conclude that there is “environmental injustice” or inequality worthy of concern.

## 4. Conclusions

EJ analyses address the hypothesis that environmental disamenities are experienced disproportionately by poor and/or minority subgroups. EJ analyses have typically used communities as the unit of analysis. While community-based approaches make sense when considering where polluting sources locate, they are less informative for analysis of national air quality rules affecting multiple sources and pollutants with long-range transport. We extend the methods and ideas in [10,17,18] and carry out a distributional benefits analysis of the HDD rule [1], which is a national rule that will impact ambient PM_2.5_ concentrations. Our distributional analysis consists in derivation of and comparisons across EJ subgroup-specific empirical distributions (over individuals) of exposure levels and/or changes in these levels resulting from the HDD rule. Using this approach, we consider a variety of characteristics of these distributions—e.g., their means or their 95th percentile values. Using the Atkinson index, a decomposable inequality index, we assess how much of the inequality across individuals is explained by an EJ subgroup characteristic (race). Finally, we make inferences about the potential effects national air quality rules may have on the inequality of exposures overall and within EJ subgroups.

We find that those subgroups that are exposed to the highest pollutant concentrations in the absence of the HDD rule will enjoy the greatest absolute reductions in exposure, on average, as a result of the rule. Because EPA rules tend to target high pollutant concentration areas, this result is likely to be representative of other national air quality rules as well. We find, however, that the HDD rule affects neither between-group inequality nor within-group inequality (as measured by the Atkinson index), because all EJ subgroups enjoy similar relative reductions in baseline exposures. Finally, inequality in exposure levels (and reductions in them) across individuals from different EJ subgroups is minor compared with the inequality among individuals within EJ subgroups.

Because changes in the corresponding health risks depend, in addition, on the subgroups’ baseline incidence rates for the health effects, and these differ across subgroups, the health risk reductions resulting from the reductions in exposure do not follow the same pattern. We find that Asian Americans and African Americans enjoy the largest absolute reductions in exposure to PM_2.5_ (out of all racial subgroups considered). However, compared to African Americans, Asian Americans receive smaller mortality risk reductions because their baseline mortality rates are much lower.

## Figures and Tables

**Table 1.** Distribution Characteristics of 2030 Projected Baseline and Control PM_2.5_ Exposures by Race and Ethnicity.

**Racial/Ethnic Subgroup**	**Mean (μg/m^3^)**	**SD (μg/m^3^)**	**Percentiles (μg/m^3^)**					**Atkinson Index**	
			**5th**	**25th**	**50th**	**75th**	**95th**	***ɛ*= 0.5**	***ɛ*= 1**
***Baseline PM_2.5_ Exposures***									

*Total Population*	*14.65*	*7.39*	*4.05*	*9.52*	*14.05*	*18.02*	*28.44*	*0.064*	*0.131*
Asian American	16.71	9.13	5.64	9.53	15.03	21.31	39.59	0.072	0.144
African American	18.13	7.50	7.42	13.22	16.99	21.47	34.47	0.042	0.085
Native American	10.22	6.97	2.47	4.43	9.17	13.74	22.61	0.106	0.207
White Hispanic	13.39	8.21	3.38	6.78	12.40	17.28	29.32	0.088	0.176
White non-Hispanic	14.07	6.45	4.16	9.61	13.89	17.22	25.35	0.054	0.113

*Within-Group Inequality*								0.060	0.123
*Between-Group Inequality*								0.004	0.009
*Ratio of Within-Group Inequality to Between-Group Inequality*								15.0	13.7

***Control Scenario PM_2.5_ Exposures***									

*Total Population*	*14.01*	*7.07*	*3.84*	*9.15*	*13.52*	*17.22*	*27.41*	*0.063*	*0.131*
Asian American	15.94	8.80	5.21	9.15	14.33	20.14	38.24	0.073	0.146
African American	17.34	7.17	7.05	12.75	16.34	20.61	32.35	0.042	0.085
Native American	9.78	6.66	2.42	4.24	8.70	13.15	21.38	0.104	0.205
White Hispanic	12.77	7.90	3.22	6.52	11.73	16.46	28.08	0.089	0.178
White non-Hispanic	13.46	6.15	4.01	9.25	13.43	16.45	23.87	0.054	0.112

*Within-Group Inequality*								0.060	0.123
*Between-Group Inequality*								0.004	0.009
*Ratio of Within-Group Inequality to Between-Group Inequality*								15.0	13.7

**Table 2.** Distribution Characteristics of 2030 Projected Absolute and Relative Reductions in PM_2.5_ Exposures by Race and Ethnicity.

**Racial/Ethnic Subgroup**	**Mean**	**SD**	**Percentiles**					**Atkinson Index**	
			**5th**	**25th**	**50th**	**75th**	**95th**	***ɛ*= 0.5**	***ɛ*= 1**
***Absolute Reductions in PM_2.5_ Exposure*(μg/m^3^)**									

*Total Population*	*0.64*	*0.39*	*0.13*	*0.38*	*0.59*	*0.83*	*1.36*	*0.088*	*0.184*
Asian American	0.77	0.41	0.25	0.45	0.69	0.93	1.48	0.068	0.137
African American	0.79	0.42	0.27	0.47	0.71	1.01	1.54	0.067	0.135
Native American	0.44	0.35	0.04	0.13	0.37	0.62	1.05	0.167	0.344
White Hispanic	0.62	0.37	0.11	0.34	0.59	0.83	1.35	0.096	0.202
White non-Hispanic	0.61	0.37	0.13	0.36	0.55	0.78	1.35	0.088	0.182

*Within-Group Inequality*								0.085	0.175
*Between-Group Inequality*								0.004	0.010
*Ratio of Within-Group Inequality to Between-Group Inequality*								21.3	17.5

***Relative Reductions in PM****_2.5_****Exposures (% of Baseline)***									

*Total Population*	*4.39*	*1.55*	*2.25*	*3.40*	*4.15*	*5.12*	*7.16*	*0.030*	*0.060*
Asian American	4.86	1.56	2.90	3.61	4.52	5.92	7.86	0.025	0.050
African American	4.33	1.38	2.55	3.40	4.09	5.12	6.90	0.024	0.048
Native American	3.97	1.87	1.44	2.68	3.77	4.89	7.68	0.054	0.108
White Hispanic	4.79	1.86	2.51	3.40	4.44	5.79	8.16	0.035	0.070
White non-Hispanic	4.22	1.43	2.08	3.34	4.10	4.89	6.90	0.028	0.058

*Within-Group Inequality*								0.029	0.059
*Between-Group Inequality*								0.001	0.002
	*Ratio of Within-Group Inequality to Between-Group Inequality*							29.0	29.5

**Table 3.** Absolute and Relative Reduction in Mean PM_2.5_ Concentrations and Incidence of All-Cause Mortality (per Million Population).

**Age/Race**	**Baseline Incidence per Million Population**	**Absolute Reduction in PM_2.5_Level (μg/m^3^)**	Relative Reduction in PM_2.5_Level *	**Absolute Reduction in Incidence per Million Population**	Relative Reduction in Incidence *
Infants (Age 0) **					
Asian American	2,907	0.71	1.2	13.7	0.7
African American	9,543	0.74	1.2	47.1	2.3
Native American	4,166	0.38	0.6	9.1	0.4
White	4,005	0.57	0.9	15.3	0.8
*Total Population*	*4,816*	*0.61*	*--*	*20.2*	--

Adults (30 64) ***					
Asian American	1,771	0.69	1.2	6.6	0.6
African American	5,183	0.73	1.2	21.7	2.0
Native American	2,587	0.40	0.7	4.7	0.4
White	3,027	0.55	0.9	9.7	0.9
*Total Population*	*3,225*	*0.58*	*--*	*11.1*	--

Elderly (65 +) ***					
Asian American	20,411	0.67	1.2	77.6	0.6
African American	39,783	0.74	1.3	170.2	1.4
Native American	25,344	0.39	0.7	52.9	0.4
White	37,945	0.54	1.0	119.7	1.0
*Total Population*	*36,863*	*0.57*	*--*	*121.4*	--

* Reductions relative to the mean reduction for the total population in the age category;

** Reductions in incidence based on the concentration-response relationship in Woodruff *et al.* [33] with *b* = 0.007339;

*** Reductions in incidence based on the concentration-response relationship in Pope *et al.* [34] with *b* = 0.005827.

**Table 4.** Distribution Characteristics of 2030 Projected Reduction in All-Cause Mortality Rate (Deaths per Million People) by Race.

**Racial/Ethnic Subgroup**	**Mean**	**SD**	**Percentiles**					**Atkinson Index**	
			**5th**	**25th**	**50th**	**75th**	**95th**	***ɛ*= 0.5**	***ɛ*= 1**
Infants (Age 0) *									

*Total Population*	*20.2*	*14.7*	*2.8*	*9.6*	*17.0*	*26.5*	*50.1*	*0.158*	*0.305*
Asian American	13.7	9.8	3.2	7.4	11.3	17.4	33.8	0.105	0.224
African American	47.1	26.6	11.3	26.2	41.4	67.2	97.0	0.084	0.175
Native American	9.1	8.4	1.3	3.0	6.8	12.2	25.4	0.170	0.343
White	15.3	10.2	2.3	7.8	13.6	20.1	35.3	0.111	0.230

*Within-Group Inequality*								0.101	0.210
*Between-Group Inequality*								0.064	0.121
*Ratio of Within-Group Inequality to Between-Group Inequality*								1.6	1.7

Adults (30 64) **									

*Total Population*	*11.1*	*7.4*	*1.6*	*5.6*	*9.8*	*14.5*	*24.8*	*0.129*	*0.261*
Asian American	6.6	3.8	1.6	4.0	5.9	8.8	12.4	0.078	0.159
African American	21.7	11.8	5.4	13.4	19.2	29.3	44.0	0.077	0.163
Native American	4.7	3.9	0.8	1.7	3.7	6.5	11.7	0.148	0.287
White	9.7	6.3	1.5	4.9	8.8	13.1	22.3	0.109	0.230

*Within-Group Inequality*								0.099	0.209
*Between-Group Inequality*								0.033	0.066
*Ratio of Within-Group Inequality to Between-Group Inequality*								3.0	3.2

Elderly (65+) **									

*Total Population*	*121.4*	*79.7*	*18.1*	*62.2*	*107.9*	*160.4*	*278.6*	*0.115*	*0.239*
Asian American	77.6	44.5	16.1	45.7	70.4	101.6	164.7	0.086	0.179
African American	170.2	92.9	41.3	97.9	157.8	231.3	328.9	0.079	0.164
Native American	52.9	47.9	6.2	14.7	40.3	75.5	158.2	0.185	0.362
White	119.7	80.7	17.3	57.9	105.5	160.1	283.5	0.114	0.238

*Within-Group Inequality*								0.109	0.226
*Between-Group Inequality*								0.008	0.018
*Ratio of Within-Group Inequality to Between-Group Inequality*								14.1	12.8

* Reductions in incidence based on the concentration-response relationship in Woodruff *et al.* [33] with *b* = 0.007339;

** Reductions in incidence based on the concentration-response relationship in Pope *et al.* [34] with *b* = 0.005827.
